# Construction of a Model of Nurse Engagement in Long-Term Care Facilities: A Moderated-Mediation Model

**DOI:** 10.3389/fpsyg.2022.798624

**Published:** 2022-06-13

**Authors:** Menglin Jiang, Jing Zeng, Xiaofang Chen, Qian Rao, Mingshu Liao

**Affiliations:** School of Nursing, Chengdu Medical College, Chengdu, China

**Keywords:** long-term care facilities, nurse engagement, job resource, emotional intelligence, organizational climate

## Abstract

**Aim:**

The aim of the study is to test a moderated mediation model that focuses on job resources mediating the relationship between organizational climate and nurse engagement in the long-term care facilities and emotional intelligence moderates this mediated relationship.

**Background:**

The shortage of nurses is a global problem, especially in the long-term care facilities. We integrated and extended past research exploring the influence of nurse engagement and constructed a model of nurse engagement in the long-term care facilities.

**Method:**

A cross-sectional survey was conducted on 494 nurses in long-term care facilities. Nurses were asked to complete a survey of nurse engagement, organizational climate, job resources, and emotional intelligence.

**Results:**

The consequence demonstrated that organizational climate increased nurse engagement directly and indirectly *via* job resources. In addition, emotional intelligence plays a moderation role between organizational climate and job resources.

**Conclusion:**

These phenomena revealed that a good organizational climate and job resources enable nurses to be more engaged in work. Nurse with high-emotional intelligence can take advantage of resources and improve their engagement.

## Introduction

The aggravation of China's aging population has brought a sharp increase in the demand for long-term care (Liu et al., 2020). Long-term care facilities, providing health care, life assistance, rehabilitation services, and even hospice care for the aged or chronically ill persons, play an essential role in mitigating the social burden and improving the quality of life of the elderly (Crayton et al., 2020). However, long-term care facilities in China are just at the fledgling stage, had existed a substantial nurse shortage. Staff shortage, an increase in patient demands, and a burdensome workload arouse the persistent decline in annual recruitment and attrition rate of nurses in long-term care facilities, which may spell a discontent balance in supply and demand. Thus, it's enlightening to explore the psychodynamic aspects such as nurse engagement in long-term care facilities to stabilize the rapid development of long-term care nursing teams and upgrade the quality of care.

Nurse engagement refers to seeking, experiencing, and adhering to meaningful work, which cannot improve the initiative and quality of nurses solely but reduce the mortality of the patients (Bargagliotti, 2012). The influencing factors of nurse engagement were summarized into six categories, including organizational climate, job resources, job demands, personal resources, professional resources, and demographic variables (Keyko et al., 2016). Then, Keyko et al. adapted the Job Demands-Resources (JD-R) model, the most commonly used theoretical model to explain the influence of engagement, and developed the Nursing Job Demands-Resources (NJD-R) model (Keyko et al., 2016). In this more refined model, the organizational climate was independent of operational level resources, highlighting the importance of organizational climate, and job resources. In addition, as nursing is an intensively emotional labor profession, nurses' emotional intelligence, an ability of emotional identification and management, is considered as a personal resource, which plays a crucial role in working. One study demonstrated that nurses with higher levels of emotional intelligence scored more highly in job engagement (Pérez-Fuentes et al., 2018).

A large and growing body of studies has conducted in-depth discussions on the relationship between organizational climate, job resources, emotional intelligence, and engagement. However, considerable uncertainty still exists about the relationship between them, what's more, there is little data that explored the interaction among the four factors, especially focusing on nurses in long-term care facilities. Therefore, based on the NJD-R model, this study explored the interactive relationship between organizational climate, job resources, emotional intelligence, and nurse engagement, in order to enrich the nurse engagement model in the long-term care facilities and provided a theoretical basis for scientific management and intervention.

### Organizational Climate and Nurse Engagement

The social exchange theory holds that the relationship of individual and organization is built on the norm of reciprocity, one party in the exchange relationship will reciprocate positively to the other party, and in doing so, this will construct an adequate atmosphere of the relationship. Organizational climate, employees' perceptions about organizational features such as the working environment (Stone et al., 2009), has a significant strike on the motivation, behavior, and attitude of organizational members (Johnston, 1971). Thus, social exchange theory provided a solid theoretical basis, suggesting an organization's investments in the light of organizational climate-like work environment, consequently perceived by nurses, would be reciprocated in the field of positive commitment and behaviors, for instance, being more engaged (Nord, 1969; Veth et al., 2018). In addition, organizational climate can vary depending on space, time, and type of employee. Labor-intensive front-line employees tend to be more sensitive to the objective material environment, while knowledgeable employees are more focused on emotional and spiritual feelings. Therefore, the organizational climate is multi-dimensional for different types of employees. On the one hand, the enthusiasm of employees is increased by fulfilling the material requirement, such as generous welfare benefits, perfect facilities, and equipment (Suliman and Aljezawi, 2018); On the contrary, the spiritual support from leaders and colleagues improve a sense of belonging (Weziak-Bialowolska et al., 2020). In short, employees with different knowledge backgrounds and in various industries perceive organizational climate differently, which can impact their engagement.

Nurses in long-term care facilities have had a heavy workload, and a high-workload environment may erode the climate that nurses perceive about their organization (Ren et al., 2020). Therefore, it is particularly significant for nurses to perceive the whole environment of long-term care facilities, which can trigger their emotional commitment, and then repay with high engagement. Based on the aforementioned, we bring forward the following hypotheses:

H1: Organizational climate is positively related to nurse engagement in long-term care facilities.

### Job Resources as a Mediator

Job resources refer to the factors in work to achieve work goals, reduce job demands, physical and psychological consumption, promote personal growth, learning and development, including social support, career opportunities, feedback, autonomy, reward, etc. (Bakker et al., 2003; Hakanen et al., 2007). Job resources are motivating, bringing in more intrinsic motives of nurses, encouraging them to invest more time and effort in the organization, boosting the organizational commitment of members (Mowday et al., 1979; Peng et al., 2021). Individuals want to accumulate and protect favorable resources in work, stimulating the work motivation to engage, and make excellent work performance. With the continuous improvement of the JD-R model, the mediating role of work resources has been revealed and confirmed. One empirical literature showed that job resources are the major mediator between employee self-management and job engagement (Breevaart et al., 2014; van Dorssen-Boog et al., 2020). Stated that job resources, such as feedback and autonomy, played a mediating role in the relationship between work engagement and health.

With further study, Keyko et al. (2016) indicated that organizational climate can indirectly affect engagement through resources. That means nurses are urged to obtain and protect resources to help them achieve their work goals. More resources nurses get in their work, the higher perceived organizational climate will be, and the higher job engagement will be witnessed (Kim and Kim, 2021). For example, social support helps nurses to enhance their perception of social and interpersonal relationships, promote the satisfaction of emotional needs, and improve their job engagement. Moreover, nurses strive to acquire and protect their resources regardless of the perceived organizational climate. Accordingly, the incentive effect of job resources facilitates higher nurse engagement. Thus, combining the aforementioned analysis and the derivation of Hypothesis 1, the following assumption is proposed:

H2: Job resources mediate the relationship between organizational climate and nurse engagement.

### Emotional Intelligence as a Moderator

Despite the fact that job resources and organizational climate are important influencing factors of nurse engagement, not all long-term care nurses are affected by both in the same way. In addition, nurses with the same resources may also exert different engagement levels, so it's necessary to consider the key to moderating variables of the relationship between job resources and engagement. Built on the perspective of social exchange theory, the process and quality of the exchange between employees and organizations would be monitored by the personal characteristics. Individuals with different emotional intelligence show unusual cognitive levels and emotional regulation abilities when acquiring resources, consequently, atypical behavior (Shore et al., 2009).

Emotional intelligence refers to the ability to manage individual and others' emotions to guide thoughts and actions (Lewis et al., 2017). Mounting evidence has presented that one's emotion may affect the perception of the surrounding environment, resource utilization, information processing, and so on, thereby affecting individual behavior (Sharma et al., 2016; Nightingale et al., 2018). This process was restricted by individual's emotional intelligence, and emotional intelligence was considered as an individual variable to regulate behavior. Nurses with higher emotional intelligence are more likely to obtain resources that offset the negative effects of burnout. Furthermore, people with high-emotional intelligence perceive a good organizational climate and establish a bidirectional dependence and mutually beneficial relationship with the organization, and maintain, consolidate, and strengthen this relationship in the way of return (Peña-Sarrionandia et al., 2015). As a link to this relationship, emotional intelligence further optimizes behavior through managing emotions and identifying work situations and content. Existing research recognized the moderating role assumed by emotional intelligence. Moreover, for nurses suffering intensive emotional labor in long-term care facilities, emotional intelligence matters a lot to them. Thus, we propose the following moderation hypothesis:

H3: Emotional intelligence plays a moderating role in the relationship between job resources and nurse engagement.

**Figure 1:**
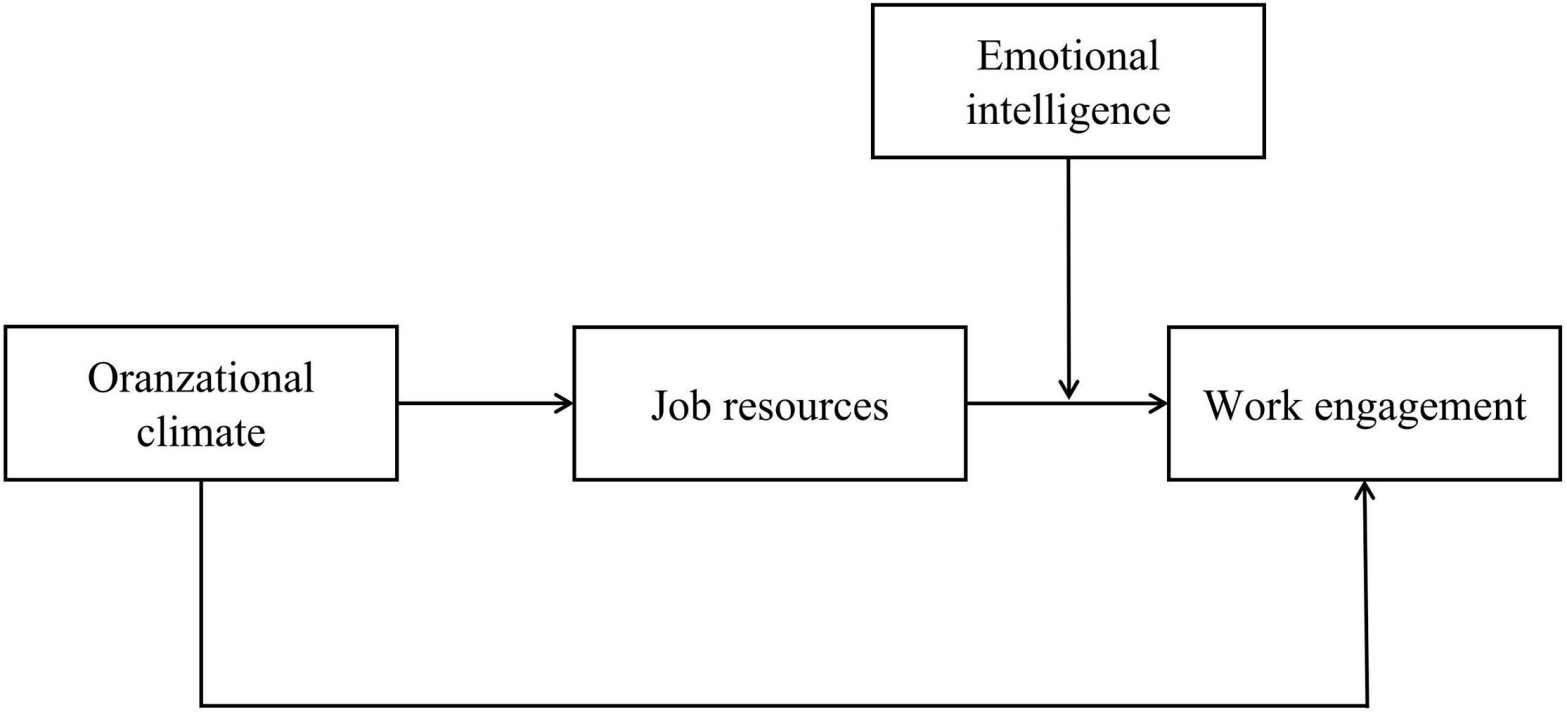
The proposed moderated mediation model.

In summary, based on the NJD-R model, this study will construct a moderated mediation model to investigate the relationship between organizational climate, job resources, emotional intelligence, and nurse engagement in long-term care facilities. The hypothetical model is shown in Figure 1.

## Materials and Methods

### Participants

The sample had been composed of 510 nurses from the three long-term care facilities in Chengdu, Sichuan, China. The convenient sampling method was employed. Inclusion criteria: (1) registered nurses in the long-term care facility such as a geriatric hospital, chronic disease hospital, and geriatric department of general Hospital; (2) the years of nursing experience >1 year; (3) those who know and agree to participate in the investigation. A total of 510 questionnaires were distributed. After excluding invalid questionnaires with more than two-thirds of the missing questions and high similarity, 494 valid questionnaires were returned. The applicable rate is 96.86%. Participants were 93% women, mainly aged 20–30 (59%) years, most of them possessed a bachelor's degree (53%) and 43% held an associate's degree. And their monthly income is located in the middle level, generally ranging from 4,001 to 6,000 yuan (42%), followed by 2,001 to 4,000 yuan (41%).

After obtaining informed consent from the nurse, all the participants were invited to fill a questionnaire anonymously. The informed consent form was issued by each participant, and each participant received a gift after finishing the questionnaire. The participants were informed that their answers would remain completely confidential and were permitted to refuse to participate in the study.

### Measures

#### Nurse Engagement

We employed the Chinese version of Utrecht work engagement scale-9 items (UWES-9), translated by Zhang and Gan (2005). This scale includes three domains: vigor, absorption, and dedication, and likert-7 scoring method was utilized. Nurse with higher scores suggesting higher engagement in work. In our study, the Cronbach's α of the scale was 0.962.

#### Emotional Intelligence

The Chinese version of Wong et al.'s Emotional Intelligence Scale translated by Kong (2017) was utilized. This scale consists of 16 items with four subscales: evaluation of emotion in oneself, evaluation of emotion in others, use of emotion, and regulation of emotion. Higher scores on the scale detect a greater level of emotional intelligence. In this study, the Cronbach's α of the scale was 0.961.

#### Job Resources

To evaluate the job resources of nurses in the long-term care facilities, we used the job resources questionnaire including four subscales: autonomy scale, social support scale, performance feedback scale, and career development opportunity scale (Gillen et al., 2002; Bakker et al., 2004). There were 22 items in total. In our study, the internal consistency of scale was good, the Cronbach's α was 0.933.

#### Organizational Climate

Organizational climate was assessed using the Chinese version of nurse's organizational climate scale complied by HeYe based on the framework of Stone for the Integrative Model of Organizational Climate (Stone et al., 2005). The scale consists of 37 items and evaluates the organizational climate of nurses in long-term care facilities from six aspects: adequate resource, team behavior, management support, quality control, human resource management, and evidence-based nursing support (Chou, 2008). In our study, the scale had excellent internal consistency, the Cronbach's α for the scale was 0.981.

### Statistical Analysis

Version 24 of the Statistical Package for the Social Sciences (SPSS) and PROCESS macro 3.3 (Hayes, 2013) were used to analyze data in this study. Before the analyses, all the continuous variables were centralized. In the beginning, descriptive statistics and correlation analysis were performed for all variables in SPSS. Next, PROCESS Model 4 (Hayes, 2013) was used to test the mediation of job resources, and the moderated mediation analysis was tested using PROCESS Model 14 (Hayes, 2013). Finally, a simple slope analysis was utilized to test whether the mediation effect of job resources was different at different levels of the moderator variable. In this study, general demographic variables such as age, working years, hospital grade, and monthly income were included as covariates. Percentile bootstrap CIs were calculated based on 5,000 samples.

### Control Variables

For the control variables, the meta-analytic findings of Keyko et al. (2016) have revealed a weak effect between age, tenure, and hospital grade. Furthermore, previous research has also controlled for age, gender, and monthly income when exploring the antecedents of nurse engagement (Yu et al., 2020). Hence, we measured the age, gender, work tenure, and periodic income to control for their potentially spurious effects.

### Common Method Bias

In this study, common method bias may occur caused by self-reporting data. We reverse the coding of scale items, anonymous answering, and counterbalancing questions in order to minimize the problem. Following the method suggested by Podsakoff et al. (2003), common method variance was tested by controlling for the effects of an unmeasured latent factor. Confirmatory factor analysis was used to test the common method bias of all self-assessment items. The results showed that the model fit was very poor, χ^2^ = 2,362.23, degrees of freedom (df) = 119, χ^2^/DF = 19.85, comparative fit index (CFI) = 0.73, goodness of fit (GFI) = 0.58, normed fit index (NFI) = 0.72, root mean square error of approximation (RMSEA) = 0.19, which indicating that there is no serious common method variance problem in this study.

## Results

### Descriptive Analysis

Controlling age, tenure, etc., general demographic variables, the descriptive statistics, and correlation analysis results show that job resources was significantly positively correlated with organizational climate, emotional intelligence, and nurse engagement. The organizational climate was positively correlated with emotional intelligence and nurse engagement. The relationship between emotional intelligence and nurse engagement was statistically positively significant. See Table 1 for details.

**Table 1:** Descriptive statistics and correlation analysis of variables.

	**M**	**SD**	**1**	**2**	**3**	**4**
1. Job resources	106.57	18.75	1			
2. Organizational climate	110.15	20.57	0.66^**^	1		
3. Emotional intelligence	15.83	2.65	0.58^**^	0.73^**^	1	
4. Nurse engagement	42.98	11.36	0.53^**^	0.65^**^	0.70^**^	1

*N = 494,*

** *p < 0.01*.

### Testing for Mediation

According to Hayes, first, we test the mediating effect of job resources on organizational climate and engagement. The mediating role of job resources was upheld. After controlling for covariates (age, tenure, monthly income, and hospital grade), organizational climate positively predicted nurse engagement, c' = 0.67, *p* < 0.001; then, organizational climate and job resources entered the regression equation at the same time, the result showed that organizational climate positively predicted job resources, *a* = 0.64, *p* < 0.001; job resources positively predicted nurse engagement, *b* = 0.19, *p* < 0.001 (see Table 2A for details). Finally, the bias corrected percentile Bootstrap method test showed that job resources play a significant mediated role between organizational climate and engagement, ab = 0.12, SE = 0.04, and 95% CI = [0.06, 0.20] (see Table 2B). Thus, job resources partially mediated the relationship between organizational climate and nurse engagement. The mediation effect accounts for 18% of the total effect. Therefore, Hypotheses 1 and 2 were validated.

**Table 2A:** Mediation effects of job resources on the relationship between organizational climate and nurse engagement.

**Dependent variable**	**Model 1**		**Model 2**		**Model 3**	
	**β**	**t**	**β**	**t**	**β**	**t**
Age	0.21	3.25^**^	0.01	0.23	0.21	3.26^**^
Tenure	−0.05	−1.34	−0.03	−0.91	−0.05	−1.19
Hospital grade	0.08	2.16^*^	−0.02	−0.58	0.08	2.30^*^
Monthly income	0.06	1.27	0.02	0.53	0.05	1.20
Organizational climate	0.67	19.39^***^	0.64	18.81^***^	0.55	12.32^***^
Job resources					0.19	4.14^***^
R2	0.38		0.44		0.47	
F	61.94^***^		73.51^***^		79.91^***^	

*N = 494*.

* *p < 0.05;*

** *p < 0.01;*

*** *p < 0.001*.

**Table 2B:** The Bootstrapping analysis of the mediating effects.

	**Effect**	**SE**	**Boot CI lower**	**Boot CI upper**	**Proportion**
Total effect	0.67	0.04	0.60	0.75	
Direct effect	0.55	0.04	0.46	0.64	82%
Indirect effect	0.12	0.04	0.06	0.20	18%

### Testing for the Moderated Mediation Model

Model 14 of PROCESS macro (Hayes, 2013) was used to test the moderating effect of emotional intelligence. Parameters of three regression equations were necessary, as shown in Table 3A. Equation 1 estimates the predictive effect of organizational climate on job resources (β = 0.64, *p* < 0.001); Equation 2 and 3 estimates the predictive effect of job resources on nurse engagement and the moderating effect of emotional intelligence on job resources and nurse engagement, respectively (β = 0.11, *p* < 0.01; β = 0.07, *p* < 0.01), and the index of the moderated mediation was 0.04, SE = 0.02, 95% CI = [0.01, 0.08], suggesting emotional intelligence moderated the association between job resources and nurse engagement.

**Table 3A:** Results of emotional intelligence moderate the mediation process.

**Dependent variable**	**Model 1**		**Model 2**	
	**β**	**t**	**β**	**t**
Age	0.01	0.23	0.18	3.10^**^
Tenure	−0.03	−0.91	−0.04	−1.20
Hospital grade	−0.02	−0.58	0.08	2.50^*^
Monthly income	0.02	0.53	0.05	1.19
Organizational climate	0.64	18.81^***^	0.26	5.17^***^
Job resources			0.11	2.60^**^
Emotional intelligence			0.49	10.46^***^
Job resources × Emotional intelligence			0.07	2.51^**^
R2	0.44		0.57	
F	73.51^***^		79.78^***^	

*N = 494*.

* *p < 0.05;*

** *p < 0.01;*

*** *p < 0.001*.

**Figure 2:**
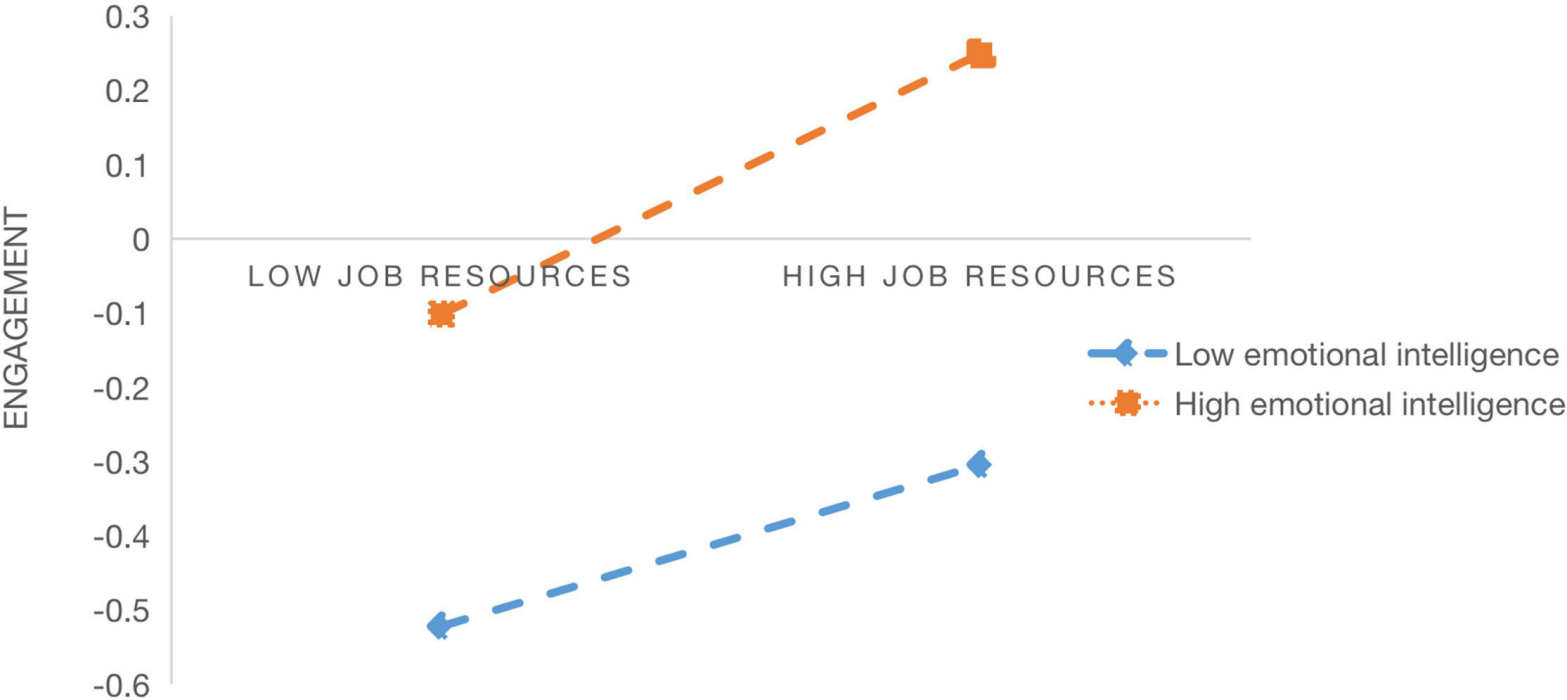
Emotional intelligence as a moderator on the relationship between job resources and work engagement.

In order to clarify the essence of the interaction effect between job resources and emotional intelligence, we divided emotional intelligence into high and low groups according to the average plus or minus a SD, conducted a simple slope analysis to explore the pattern of the moderating effect see Figure 2. The results indicated that only in the nurses with high-emotional intelligence, job resources were positively correlated with nurse engagement (Bsimple = 0.17, *p* < 0.001). Moreover, the indirect effect was significant for nurses with higher emotional intelligence (index = 0.11, BootSE = 0.04, 95% CI = [0.05, 0.19]), and for those with lower emotional intelligence, the indirect effect was not significant (index = 0.03, BootSE = 0.03, 95% CI = [−0.03, 0.09]), see Table 3B. Overall, these results suggested that emotional intelligence moderated the relationship between organizational climate and nurse engagement via job resources, Hypothesis 3 was supported.

**Table 3B:** Conditional indirect effect of emotional intelligence when job resources mediated between organizational climate and nurse engagement.

**Mediator**	**Emotional intelligence**	**Effect**	**BootSE**	**BootLLCI**	**BootULCI**
Job resources	M-SD	0.03	0.03	−0.03	0.09
	M	0.07	0.03	0.02	0.13
	M+SD	0.11	0.04	0.05	0.19

## Discussion

This study revealed the relationship between organizational climate and nurse engagement and clarified how the underlying mechanism of organizational climate predicted nurse engagement. First, it illustrated how the organizational climate affected engagement, including its direct influence and the mediating role of job resources. Second, it analyzed “how it functions more.” That is, the mediating effect of job resources was distinguished as being affected by different levels of emotional intelligence.

In our study, the results show that the organizational climate was a facilitator of nurse engagement. High levels of organizational climate can increase nurse engagement in long-term care facilities; these results support our hypothesis. According to our survey, the engagement of long-term care nurses was at a medium level and there was great variability among individuals. That is probably caused by: initially, long-term care nurses perceived a bad organizational climate attributed to poor-medical service conditions of long-term care institutions (Hsieh and Chen, 2017). Furthermore, older people's physical functions gradually degenerate with age, the vision, hearing, and cognition are impaired, increasing the difficulty of nurse–patient communication. Good communication with patients is a prerequisite for a satisfactory relationship, which may be a particularly important factor for enhancing nurses' perception of organizational climate. In addition, geriatric nursing education started late in China. Most of the long-term care nurses have not received systematic and standardized care skills training. That's why they often feel frustrated in long-term care works (Hsieh and Chen, 2017). Previous studies have examined that the work environment and interpersonal relationships can impact nurse engagement (Manning, 2016). In this study, organizational climate acts as an important factor influencing nurse engagement. In this sense, nurse managers should give due consideration to the long-term impact of the work environment on nurses and keep an eye on the construction of organizational culture and forge a wholesome organizational climate for the nurses.

What's more, we extend the NJD-R theoretical model and verify that job resources such as social support, job feedback, and career development opportunities play a mediating role in the relationship between organizational climate and engagement. In other words, when the long-term care nurse perceived a poor-organizational climate, they would seek or protect advantageous resources to offset negative effects such as emotional exhaustion caused by job demands, which make a balance between “burnout” and “motivation” to maintain their engagement level. The more work resources nurses obtained, the needs they were satisfied with, the better their perception of organizational climate accordingly (Yulita et al., 2020). To put it simply, job resources liked high-social support, bright prospects for development may make for a cordial interpersonal relationship and resources security, which forge a healthy organizational climate for nurses. Nurses hope for and derive pleasure from support and relation in work. Consequently, they may be in a position to possess higher engagement by taking pride in their work and enjoying it (Mukaihata et al., 2020). Thus, awareness could be raised among supervisors on providing adequate resources to nurse, in order to promote the personal growth and development of nurses.

More importantly, this study found that emotional intelligence moderated the latter half of the process of organizational climate–job resources–engagement. Specifically, nurses with high-emotional intelligence were apt to accept the organizational climate positively by actively acquiring more job resources. Then, they would be more vigorous and engaged at work. This suggested that emotional intelligence enhances the mediating effect of job resources on organizational climate and engagement, which supports the affective events theory (Carlson et al., 2011). However, the emotion itself cannot increase job resources but is achieved by increasing the feasibility of individual behavior and more cognitive processes. Since emotional intelligence is individualized, the abilities to comprehend, adjust, and use emotions of everyone were different and showed personalized behavior. What the mean is, nurses with high-emotional intelligence were more likely to find the most effective ways of mobilizing positive emotions and quickly respond to events and circumstances of the work. And sensitivity to the environment makes it easier for them to attain resources, which invites them to get into the swing of their work.

## Limitations and Research Perspectives

There are several limitations to this study. First, since the data were derived from a cross-sectional survey, we could not infer or verify the causal relationships among variables. Second, we did not focus on the gain spiral benefits of engagement with resources and organizational climate, a longitudinal design could be used in future studies. Third, our study only investigated nurses in Sichuan Province, which limited the generalization of the research results. Future research will try to sample from China.

## Conclusion

This study constructed a moderated mediation model to explore the interrelationship and mechanism of influence of nurse engagement in long-term care facilities and serve as a theoretical basis for improving nurse engagement and stabilizing the development of the long-term care team.

## Data Availability Statement

The original contributions presented in the study are included in the article/supplementary material, further inquiries can be directed to the corresponding author.

## Ethics Statement

This study was reviewed and approved by The First Affiliated Hospital of Chengdu Medical College (No. 2020CYFYIRB-BA-113). Written informed consent was obtained from all participants for their participation in this study.

## Author Contributions

MJ was mainly involved in all aspects of the study and preparation of the manuscript. JZ was engaged with the design of the study and preparation of the manuscript. XC was involved with statistical analysis of data. ML and QR were involved with chart making. All authors contributed to the article and approved the submitted version.

## Conflict of Interest

The authors declare that the research was conducted in the absence of any commercial or financial relationships that could be construed as a potential conflict of interest.

## Publisher's Note

All claims expressed in this article are solely those of the authors and do not necessarily represent those of their affiliated organizations, or those of the publisher, the editors and the reviewers. Any product that may be evaluated in this article, or claim that may be made by its manufacturer, is not guaranteed or endorsed by the publisher.
